# Spectrophotometric Determination of Cu^2+^ and Monitoring of Hg^2+^ and Ni^2+^ in some Iranian Vegetables Using 6-(2-Naphthyl)-2, 3-Dihydro-as-triazine-3-thione 

**Published:** 2013

**Authors:** Fazel Shamsa, Malehe Barazande Tehrani, Hamid Mehravar, Elaheh Mohammadi

**Affiliations:** *Department of Pharmaceutical Chemistry, Faculty of Pharmacy and Pharmaceutical Sciences Research Center, Tehran University of Medical Sciences, Tehran, Iran. *

**Keywords:** Copper in vegetables, Determination, Spectrophotometry, NDTT, Liquid-liquid extraction.

## Abstract

Recently, 6-(2-naphthyl)-2, 3-dihydro-as-triazine-3-thione (NDTT) was synthesized in laboratory and used successfully for the spectrophotometric determination of nanogram levels of Cu^2+^ in aqueous solution. This reagent forms a specific red complex with Cu^2+^ ions after the extraction by chloroform at alkaline pH. The absorption of the complex in the UV region (313 nm) is about 8 times as strong as in the visible one (510 nm). Mercury and nickel ions form yellow complexes with NDTT under the same conditions which interfere in the UV region and without effect on Cu (II) absorbance in the visible region. The studied vegetables include *Mentha pipereta *L., *Anethum graveolens *L., *Beta vulgaris *L., *Coriandrum sativum*, *Petroselinum hortense *H., *Ocimum basilicum *L., *Spinacia oleracea *L., *Lactuca sativa *L., and *Brassica oleracea *L.

## Introduction

Copper is a mineral that nowadays poses few problems. It is widely distributed, as a component of various enzymes in foodstuffs of all kinds, at levels between 1 and 5 ppm. Milk is notably low in copper, at around 2 ppm, and mammalian liver is exceptionally high, at around 80 ppm. The daily intake in normal adult diets is between 1 and 3 mg, which roughly corresponds to the intake level recommended by most authorities (1). Copper is an essential element in the nutrition of animals and human. It acts as a cofactor in numerous enzymes and plays an important role in protein and cell division. It exerts a crucial influence on the maintenance of cell membrane stability and in the function of immune system. Meanwhile, as a pollutant, copper is of particular concern, due to its high toxicity on aquatic organisms (1-3). 

 Determination of trace amounts of copper has received considerable attention and various methods have been developed for this purpose (2-6). These methods are time-consuming, not sufficiently sensitive or narrow range pH-dependent. Therefore, developing specific and sensitive methods without being pH-dependent which rapidly and conveniently detect and determine copper in real samples seems to be desirable (4, 5). 

Previously, 6-phenyl-2, 3- dihydro-as-triazine-3-thione (PDTT) was reported to form a specific red complex with Cu^2+^, which is easily extractable with chloroform over a wide range of pH. Despite being simple, this method suffers from low sensitivity, in a way that the molar absorbance of the complex is 5.0 × 10^3^ (6). In a previous investigation, we have reported the synthesis of 6-(2-naphtyl)-2, 3-dihydro-as-triazine-3-thione (NDTT) as a new sensitive and specific reagent for determining Cu^2+^ (7). The purpose of the modification was to increase the PDTT sensitivity in determining Cu^2+^, Hg^2+^, and Ni^2+^. The preparation of NDTT was conducted by a one-step synthesis of the as-triazine system (8).

The analysis of Cu^2+^ in solution by NDTT could be performed easily and in the presence of many cations and anions (7). Only Hg^2+^, Ni^2+^ and Pd^2+^ form complexes with NDDT. Hg^2+^ and Ni^2+^ present natural products in ultra-trace quantities compared with copper. Therefore, the analysis of Cu^2+^ in vegetable through NDTT could be considered specific by this procedure.

## Experimental

*Apparatus and reagents*

A Shimadzu 160A UV-VIS spectrophotometer with 1.0 cm quartz cell was used for all absorbance measurements and Atomic Absorption Varian 220 was employed in this study. Sodium hydroxide solution (4 M), tartaric acid solution (2 M) and NDTT in NaOH 4M (1 mg/5 mL) were used. Fresh NDTT/NaOH solution should be used. NDTT reagent itself was prepared according to the reported procedure (6). All reagents used are of analytical reagent grade, unless otherwise stated.

*Vegetables under study*

All the vegetables were bought from Tehran daily markets and then approved by Dr. Sh. Rezazadeh. The edible portions of the vegetable under study (1-2 Kg) were separated, weighed, washed thoroughly with distilled water, and left to be dried at room temperature. The weight of the dried samples were recorded and the percentages of the dried to fresh samples were calculated (Table 1). The studied vegetables include: *Mentha piperita *L., *Allium *L., *Anethum graveolens *L., *Beta vulgaris *L., *Petroselinum hortense *H., *Coriandrum sativum*, *Ocimum basilicum *L., *Spinacia oleracea *L., *Lactuca sativa *L. and *Brassica oleracea *L.

**Table 1:** Weight and percentage of the dried vegetables obtained from the corresponding fresh clean samples

**Vegetable scientific name**	**Weight of Vegetable (gm)**		%D/F*
	**Fresh**	**Dried**
*Mentha piperita *L.	360	70	19.4
*Allium *L.	1100	75	6.8
*Anethum graveolens *L.	415	50	12.0
*Beta vulgaris *L.	500	30	6.0
*Petroselinum hortense *H.	450	75	16.7
*Coriandrum sativum*	550	55	10.0
*Ocimum basilicum *L.	600	65	10.8
*Spinacia oleracea *L.	900	80	8.9
*Lactuca sativa *L.	1160	55	4.74
*Brassica oleracea *L.	1100	85	7.73

*D/F = dried/fresh

*Stock solution of copper nitrate*

 Pure elemental copper (exactly 0.5 g) was dissolved in hot con. HNO_3_. After cooling, 50 mL of HNO_3_ (1:1) was added and the volume was adjusted to 500 mL by distilled water. Solutions with lower concentrations were prepared by proper dilutions.

*Calibration curve for determining Cu* ^2+^

A mixture of 1-20 μg Cu^2+^, NDTT solution (2 mL of 1 mg / 5 mL) and tartaric acid (1 mL) in a 100 mL separatory funnel was shaken thoroughly. The resulted complex was extracted with 4, 3 and 2 mL of CHCl_3_. The extracts were collected in a 10 mL volumetric flask and adjusted to volume with CHCl_3_. The absorbance of the extracts was measured at both UV (313 nm) and visible (510 nm) region vs. a blank. A calibration curve was plotted for the amount of Cu^2+^ against the relative absorbance. This curve was used to determine the vegetable Cu^2+^ content.

**Table 2:** Percentage of ashes obtained from different dried vegetables under study (2.0 gm) after wet digestion at 600°C

**Vegetable scientific name**	**Ash**		
	**Wt. (gm)**	**%**	**%in relative to fresh sample**
*Mentha piperita *L.	0.19	9.55	2.0
*Allium *L.	0.412	20.6	1.4
*Anethum graveolens *L.	0.32	16.0	1.92
*Beta vulgaris *L.	0.49	24.5	1.47
*Petroselinum hortense *H.	0.225	11.25	1.88
*Coriandrum sativum*	0.303	15.15	1.52
*Ocimum basilicum *L.	0.308	15.4	1.66
*Spinacia oleracea *L.	0.275	13.74	1.22
*Lactuca sativa *L.	0.241	12.06	0.57
*Brassica oleracea *L.	0.100	5.0	0.39

*Vegetable sample preparation for Cu* ^2+^ *analysis*

A portion of the dried vegetable (exactly 2.0 g) was transferred to a crucible and soaked with distilled water (5 mL). The crucible was left for 1 h at 150°C followed by 4 h at 600°C for ignition. The remained ash was dissolved in con. HNO_3_ (1 mL) followed by distilled water (15 mL), filtered (if necessary), and neutralized by NaOH 4 M. The solution was made to volume in a 25 mL volumetric flask.

*Preparation of the blank solution*

Distilled water (15 mL) in a crucible was left for 1 h at 100°C and for 4 h at 600°C. After cooling, con. HNO_3_ (1 mL) and dis. H_2_O (15 mL) were added and neutralized in a beaker by NaOH 4M. This solution was transferred to a volumetric flask and made to volume (25 mL) by dis. H_2_O. A 5 mL portion of this solution was transferred to a separatory funnel, followed by tartaric acid (1 mL), NDTT solution (2 mL) and mixed thoroughly. The complex was extracted by 4, 3 and 2 mL of CHCl_3_, transferred to a 10 mL volumetric flask and made to volume. The absorption of this solution was measured against CHCl_3_. This solution was colorless with no absorption at 510 nm, but at 313 nm, it showed insignificant absorption (< 0.02). This solution was used as a blank in determining Cu^2+^ content of different vegetables.

*General procedure for the determination of Cu* ^2+^ *in vegetables*

A portion (5 mL) of the processed solution of vegetable was transferred to a separatory funnel. Tartaric acid solution 1 M (1 mL), followed by NDTT solution (2 mL) were added and mixed. The extraction of Cu-NDTT complex was performed by 4, 3 and 2 mL of CHCl_3_, collected in a 10 mL volumetric flask and made to volume by CHCl_3_. The absorbance at UV (313 nm) and visible (510 nm) was recorded. Using a calibration curve constructed for pure Cu^2+^, the μg amount of Cu^2+^ in 5 mL of the processed solution of the vegetable was obtained, and transformed to Cu^2+^ in the dried and fresh samples (Tables 3 and 4) by proper calculations.

## Results and Discussion

To perform the proposed procedure for determining copper in vegetables, primarily a portion of the edible part was weighed and dried in room temperature. The ratio of the dried to fresh samples was registered (Table 1). As shown in this table, Lactuca sativa L. contains the highest percent of water (95.26%), where Mentha piperita contains the lowest one (70.6%). For preparing the vegetable for analysis, primarily, the dried vegetable (2.0 g) was soaked with distilled water, dried in 150°C and followed by ignition at 600°C for 4 h. Since the vegetable cellulose is not hard, the wet-digestion was undertaken. A white ash which was soluble in concentrated HNO_3_ was obtained, otherwise the solution was filtered. After neutralizing by NaOH 4M, the volume was made to 25 in a volumetric flask. A portion of this solution (5 mL) was used for the analysis of Cu^2+^ and monitoring of other cations. In all cases, the chloroform layer was red and similar to a pure Cu^2+^ solution (except for anethum which was pale orange) (Table 3).

**Table 3:** Absorbances, λ_max_, and the color of the chloroformic layers of Cu-NDTT complexes of different vegetables**.**

**Vegetable scientific name**	**Absorbance/W.L.**		**Ratio UV/Vis Absorbance**	**Color of CHCl**_3_ **layer**
	UV	Visible	
*Mentha piperita *L.	0.448/313	0.053/510	8.45	Red
*Allium *L.	0.528/314	0.062/511	8.52	Red
*Anethum graveolens *L.	0.306/313	0.058/509	5.28	Pale orange
*Beta vulgaris *L.	0.599/313	0.075/510	8.00	Red
*Petroselinum hortense *H.	0.463/313	0.053/509	8.74	Red
*Coriandrum sativum*	0.467/313	0.057/509	8.2	Red
*Ocimum basilicum *L.	0.421/313	0.049/507	8.6	Red
*Spinacia oleracea *L.	0.587/314	0.064/510	9.7	Red
*Lactuca sativa *L.	0.529/314	0.034/510	15.6	Red
*Brassica oleracea *L.	0.276/313	0.033/510	8.36	Red

Therefore, it is possible to detect Cu^2+^ in vegetables easily, as NDTT formed a red complex only with Cu^2+^. The absorption spectra of the complex in chloroform were recorded and the absorbance at UV and visible regions were registered. Using a calibration curve, it was possible to find the Cu^2+^ content in 5 mL of solution. By proper calculations, the content of Cu^2+^/Kg of fresh vegetable was obtained (Table 4). 

**Table 4:** Cu^2+^ content in different steps of the analytical procedure

**Vegetable scientific name**	**Absorbance at UV λ**_max_	**Cu** ^2+^ **content**		
		**In processed solution μg/5 mL**	In 2 g dried sample (μg)*	In 1 Kg fresh sample (mg)** (ppm)
*Mentha piperita *L.	0.448	8.1	40.5	3.94
*Allium *L.	0.528	9.5	47.5	1.62
*Anethum graveolens *L.	0.306	5.51	27.5	1.65
*Beta vulgaris *L.	0.599	10.8	54.0	1.62
*Petroselinum hortense *H.	0.463	8.3	41.5	3.47
*Coriandrum sativum*	0.467	8.4	42.0	2.1
*Ocimum basilicum *L.	0.421	7.6	38.0	2.05
*Spinacia oleracea *L.	0.587	10.6	53.0	2.36
*Lactuca sativa *L.	0.529	5.0	25.0	0.593
*Brassica oleracea *L.	0.276	5.0	25.0	0.966

*Cu^2+^/2 g dried sample is obtained from the Cu^2+^/5 mL test solution multiplied by 5.

**Cu^2+^(mg)/Kg fresh sample = Cu^2+^(μg)/2 g dried sample × 0.5 × %D/F ÷ 100. ***Cu^2+^ content was obtained from the visible absorbance.

It was found that Mentha piperita L. contains the highest amount of Cu^2+^ (3.94 ppm of fresh vegetable) and Lactuca sativa L. contains the lowest amount (0.593 ppm of fresh vegetable) among the studied vegetables. To confirm the applicability of the proposed method for determining Cu^2+^ in vegetables, the samples were analyzed by atomic absorption method and the results are shown in Table 5. In all cases, there was insignificant difference between the proposed method and the atomic absorption analysis. The difference ranges from 2.5 to 11.1%. The cations Hg^2+^ and Ni^2+^ formed yellow complexes with NDTT (9, 10), hence, the absence of these two cations could be concluded as the chloroformic layer was red except in the case of Anethum graveolens L. which appeared pale orange. Therefore, Hg^2+^ and Ni^2+^ were either absent or present in non-detectable level by NDTT. Finally, it is concluded that the developed spectrophotometric procedure for determining Cu^2+^ in vegetables using NDTT is very simple, fast and specific.

**Table 5:** Copper content (ppm) of fresh vegetables found by the proposed method (NDTT) and atomic absorption method (n = 5).

**Sample**	**NDTT method**	**Atomic absorption**	**% difference**
*Mentha piperita *L.	3.94 ± 0.6	4.2 ± 0.72	6.5
*Allium *L.	1.62 ± 0.32	1.73 ± 0.35	6.8
*Anethum graveolens *L.	1.65 ± 0.35	1.72 ± 0.4	4.2
*Beta vugaris *L.	1.62 ± 0.28	1.8 ± 0.3	11.1
*Petroselinum hortense *H	3.47 ± 0.72	3.67 ± 0.68	5.8
*Coriandrum sativum*	2.1 ± 0.43	2.2 ± 0.61	4.8
*Ocimum basilicum *L.	2.05 ±0.41	2.0 ± 0.52	2.5
*Spinacia oleracea *L.	2.36 ± 0.46	2.30 ± 0.41	2.5
*Lactuca sativa *L.	0.593 ± 0.08	0.63 ± 0.07	5.0
*Brassica oleracea *L.	0.966 ± 0.11	1.02 ± 0.12	5.6
